# Gait asymmetry in spatiotemporal and kinetic variables does not increase running-related injury risk in lower limbs: a secondary analysis of a randomised trial including 800+ recreational runners

**DOI:** 10.1136/bmjsem-2023-001787

**Published:** 2024-01-05

**Authors:** Laurent Malisoux, Paul Gette, Nicolas Delattre, Axel Urhausen, Daniel Theisen

**Affiliations:** 1 Department of Precision Health, Luxembourg Institute of Health, Strassen, Luxembourg; 2 Movement Sciences Department, Decathlon SportsLab Research and Development, Villeneuve d'Ascq, Nord, France; 3 Sports Clinic, Centre Hospitalier de Luxembourg, Luxembourg, Luxembourg; 4 Luxembourg Institute of Research in Orthopedics Sports Medicine and Science, Luxembourg, Luxembourg; 5 ALAN – Maladies Rares Luxembourg, Luxembourg, Luxembourg

**Keywords:** Biomechanics, Running, Prevention, Injury

## Abstract

**Objective:**

To investigate asymmetry in spatiotemporal and kinetic variables in 800+ recreational runners, identify determinants of asymmetry, investigate if asymmetry is related to greater running injury risk and compare spatiotemporal and kinetic variables between the involved and uninvolved limb at baseline in runners having sustained an injury during follow-up.

**Methods:**

836 healthy recreational runners (38.6% women) were tested on an instrumented treadmill at their preferred running speed at baseline and followed up for 6 months. From ground reaction force recordings, spatiotemporal and kinetic variables were derived for each lower limb. The Symmetry Index was computed for each variable. Correlations and multiple regression analyses were performed to identify potential determinants of asymmetry. Cox regression analyses investigated the association between asymmetry and injury risk. Analysis of variance for repeated measures was used to compare the involved and uninvolved limbs in runners who had sustained injuries during follow-up.

**Results:**

107 participants reported at least one running-related injury. Leg length discrepancy and fat mass were the most common determinants of asymmetry, but all correlation coefficients were negligible (0.01–0.13) and explained variance was very low (multivariable-adjusted R^2^<0.01–0.03). Greater asymmetry for flight time and peak breaking force was associated with lower injury risk (HR (95% CI): 0.80 (0.64 to 0.99) and 0.96 (0.93 to 0.98), respectively). No between-limb differences were observed in runners having sustained an injury.

**Conclusion:**

Gait asymmetry was not associated with higher injury risk for investigated spatiotemporal and kinetic variables.

**Trial registration number:**

NCT03115437.

**WHAT IS ALREADY KNOWN ON THIS TOPIC**

- Gait asymmetries in lower limbs during running might generate differences in mechanical stress exposure and injury risk.
- There is currently no consensus on ‘normal’ levels of asymmetry in running specific populations.
- The association between running biomechanics asymmetry and injury risk in running is currently not established.

**WHAT THIS STUDY ADDS**

- The magnitude of asymmetry varies considerably across the spatiotemporal and kinetic variables investigated in recreational runners.
- There was a large between-subject variability in asymmetry for most spatiotemporal and kinetic variables investigated.
- Asymmetry was not associated with higher injury risk for spatiotemporal and kinetic variables investigated.
- No difference in the spatiotemporal and kinetic variables was observed at baseline between the involved and uninvolved limbs in runners who had sustained an injury during follow-up.

**HOW THIS STUDY MIGHT AFFECT RESEARCH, PRACTICE OR POLICY**

- The search for gait asymmetry in the lower limbs in healthy recreational runners as an approach for primary prevention is not supported by scientific evidence.
- Given the large variation in asymmetry levels across the variables investigated, applying a threshold to determine ‘acceptable’ or ‘normal’ asymmetry levels appears challenging.

## Introduction

One in two runners is estimated to sustain a musculoskeletal injury in a given year.^1^ Although the origin of running-related injury is complex and multifactorial,^2^ biomechanical factors may play a key role. Asymmetries in lower limb kinetics during running might generate differences in mechanical stress exposure and injury risk. Indeed, based on a framework for injury aetiology,^2^ the development of a musculoskeletal injury in one limb could result from greater loading, lower loading tolerance or some combination of the two factors. This may explain why side-to-side differences regarding anthropometrics, strength and gait biomechanics, usually defined as interlimb asymmetry, are often studied in a clinical and scientific context, with greater asymmetry typically considered a risk factor for pathology.^3 4^

There is currently no data on ‘normal’ levels of gait asymmetry in a running-specific population, especially across running speeds.^5 6^ Asymmetry in common variables associated with injury risk was assessed across different running speeds, and levels of asymmetry ranged from a <3° difference in joint kinematics to up to 40% difference in kinetics.^7^ However, as the study included both runners and non-running specific athletes, the levels of asymmetry may not be representative for runners. Several factors, most notably running speed,^7 8^ have been proposed to affect running biomechanics and the degree of asymmetry between limbs. However, only a few studies have focused on the determinants of gait asymmetry in running.

How gait asymmetry relates to running injury risk has mainly been studied retrospectively.^3 9 10^ However, retrospective designs do not allow determining whether asymmetry is the cause or the effect of injury. Symptomatic runners may alter their running mechanics and exacerbate lower limb asymmetry, which may, in turn, lead to mechanical overload and injury. Only two prospective studies have related gait asymmetry and injury risk.^5 11^ In cross-country runners followed over 12 weeks, there were no differences in asymmetry in kinematic, kinetic and spatiotemporal variables between injured runners and those that remained injury free, but the sample size was very small (n=22).^5^ In 210 novice runners engaged in a 9-week training programme, the asymmetry in spatiotemporal and kinetic variables was unrelated to injury risk.^11^

Understanding the relationship between asymmetry and injury risk could be relevant for clinicians and researchers as runners at risk could be identified by comparing differences in biomechanical variables from both legs, and preventive measures could be introduced to reduce asymmetry, including running technique interventions to reduce load imbalances, when a link to previous injury is deemed clinically plausible.^11^ Similarly, understanding the main determinants explaining the intersubject variability of asymmetry may help identify runners at risk, provided asymmetry relates to injury risk. Therefore, the objectives of this study were to (1) describe the magnitude of asymmetry in spatiotemporal and kinetic variables in 800+ recreational runners, (2) identify the determinants of asymmetry, (3) investigate if asymmetry increases running-related injury risk and (4) compare spatiotemporal and kinetic variables at baseline between the involved and uninvolved limb in runners having sustained an injury during follow-up.

## Methods

### Study design

This is a secondary analysis of a participant and assessor-blinded randomised trial (ClinicalTrials.gov, NCT03115437, 11 April 2017) with a biomechanical running analysis at baseline and a 6-month follow-up on running exposure and injury risk, comparing two running shoe prototypes with different cushioning properties.^12^ Reporting of the study followed the Consolidated Standards of Reporting Trials statement.^13^

### Patient involvement statement

Study participants were not involved in the current research’s design, conduct, interpretation or translation.

### Participants Injuries

Recreational runners were recruited from September 2017 to January 2018 from Luxembourg and the neighbouring regions. Volunteers in good health, aged 18–65 years and capable of performing 15 min of consecutive running were included in the study. Exclusion criteria were any running-impeding injury (ie, any musculoskeletal pain that causes a restriction (distance, speed or duration) or stoppage of running practice) over the previous month, medical contraindication to performing running activity, prior (<12 months) lower limb or back surgery and use of orthopaedic insoles for running. Furthermore, participants were excluded if less than five steps per limb without a double support phase were available for analysis.

### Baseline evaluations

Volunteers completed an online questionnaire regarding running experience and previous injury. During the subsequent visit to the laboratory, participants randomly received one of two running shoe versions specifically designed for the parent trial^12 14^ differing only in their cushioning properties.^14 15^ Height, body mass, fat mass proportion (Tanita SC-240 MA) and leg length^12^ were measured before the running test performed in the randomly allocated shoe version on an instrumented treadmill (M-Gait, MotekForcelink Amsterdam, the Netherlands). The test comprised a 3 min warm-up and a 10 min run at the self-declared preferred running speed (hereafter referred to as running speed). The recording was performed over the last 2 min of the test. Three-dimensional ground reaction force (GRF) data were sampled at 2 kHz and filtered using a cut-off frequency of 30 Hz with a bidirectional (zero-lag) second-order Butterworth low-pass filter.

### Follow-up procedures

The primary outcome was the first running-related overuse injury occurring during the 6-month follow-up, defined as a running-related musculoskeletal pain in either of the two lower limbs that causes a restriction (distance, speed or duration) or stoppage of running for at least 7 days (adapted from Yamato *et al*).^16^ The participants were instructed to self-report all their sports activities, running session characteristics and any adverse events (injuries, pain and illnesses) during any given session on a dedicated electronic platform on a daily basis (or minimum once a week) throughout the prospective follow-up.^17 18^ The research team checked all injury data for coherence and contacted the participants for a final check of all recordings.

### Signal processing

GRF data were processed using a custom programme written in Matlab (Matlab R2014a, MathWorks, USA). First, initial contact and toe-off events were identified by vertical force exceeding and falling below a 20 N threshold, respectively. The following spatiotemporal and kinetic variables were computed: step length, contact time, flight time, duty factor, vertical oscillation of the body centre of mass, vertical stiffness, peak vertical GRF, peak braking force and peak propulsive force. Detailed information on data processing has previously been published.^15 19^

Previous work has demonstrated the relevance of frequency-domain analysis in investigating the relationship between impact forces and injury risk.^20 21^ Thus, we decomposed the vertical GRF signal for each stance phase into the frequency domain and separated the high-frequency and low-frequency components of the signal using 10 Hz as the cut-off value.^20 22 23^ The high-frequency component was then recomposed into the time domain using the inverse Fourier transform to form the new signal and compute the variables of interest.^21^ Impact peak force was defined as the maximal value of the high-frequency signal, and time to impact peak force was defined as the time between initial contact and impact peak force. Vertical instantaneous loading rate was the maximal slope of the high-frequency signal during the same period. Vertical average loading rate was calculated as the average slope in the high-frequency signal between 20% and 80% of the period between initial contact and impact peak force. The kinetic variables were normalised to the participant’s body weight. Discrete variables were calculated for each gait cycle of the 2 min recording and averaged per subject’s limb.

Gait asymmetry was assessed in each runner for spatiotemporal and kinetic variables using the Symmetry Index (%)^7 9^:

$$SymmetryIndex=\frac{|X_{r}-X_{l}|}{0.5\left(X_{r}+X_{l}\right)}\times 100$$

where *X*_*r*_ and *X*_*l*_ are the average values of the variable in the right and left limbs, respectively, a higher value indicates a higher level of asymmetry.

### Statistical analyses

All analyses were performed using STATA/SE V.15. The potential determinants of asymmetry were investigated in two steps. These determinants included sex, age, height, body mass, fat mass proportion, running experience, running regularity (number of months with weekly running practice over the previous year), previous injury, shoe version (ie, hard or soft version), running speed and leg length asymmetry (ie, Symmetry Index). First, Pearson and point-biserial correlation coefficients were computed for continuous and dichotomous variables to explore the relationship between each potential determinant and asymmetry for each biomechanical variable. Second, multiple linear regressions (one for each biomechanical variable) with forward selection (p values<0.10) were performed to identify determinants of asymmetry. The model fit was inspected using the adjusted R^2^. We checked for multicollinearity in the final model using the variance inflation factor, with a threshold set to 10.

The association between asymmetry and injury risk was investigated using competing risks proportional hazards regression models, which estimate the subdistribution hazard (subhazard rate ratio).^24^ Time at risk was defined as hours of running practice self-reported by the participant and cumulated from the baseline evaluation date to the date of injury or censoring. The assumption of proportional hazards was evaluated using log-minus-log plots and Schoenfeld residuals. Model 1 presents the crude estimates of subhazard rate ratios and their 95% CI for each predictor (ie, Symmetry Index) separately. Model 2 adjusted for potential confounders. Model 3 is model 2 with stepwise forward selection (p values<0.10) of the biomechanical variables. Model 4 is model 3 stratified by shoe version, as shoe cushioning was associated with injury risk in the parent trial.^14^

Analysis of variance for repeated measures was used to compare the means between the involved and uninvolved limbs in runners who had sustained an injury during the follow-up period. The Benjamini-Hochberg procedure was applied to correct p values for multiple statistical comparisons.^25^ Effect sizes were reported using Cohen’s d and were interpreted as negligible (0 to <0.15), small (0.15 to <0.40), medium (0.40 to <0.75), large (0.75 to <1.10) and very large (≥1.10).^26^ The significance level was set at p<0.05.

## Results

### Participants

In total, 874 volunteers fulfilled the inclusion criteria and performed the running test at baseline. In total, 38 participants were excluded from the analyses because they did not upload any training data (n=19), were diagnosed with arthrosis during follow-up (n=3), reported untrustworthy training data (n=2), used orthopaedic insoles (n=1), had another health issue (n=1) or had less than five steps without double support phase per limb (n=12) (online supplemental material S1).

10.1136/bmjsem-2023-001787.supp1 Supplementary data

**Figure 1:**
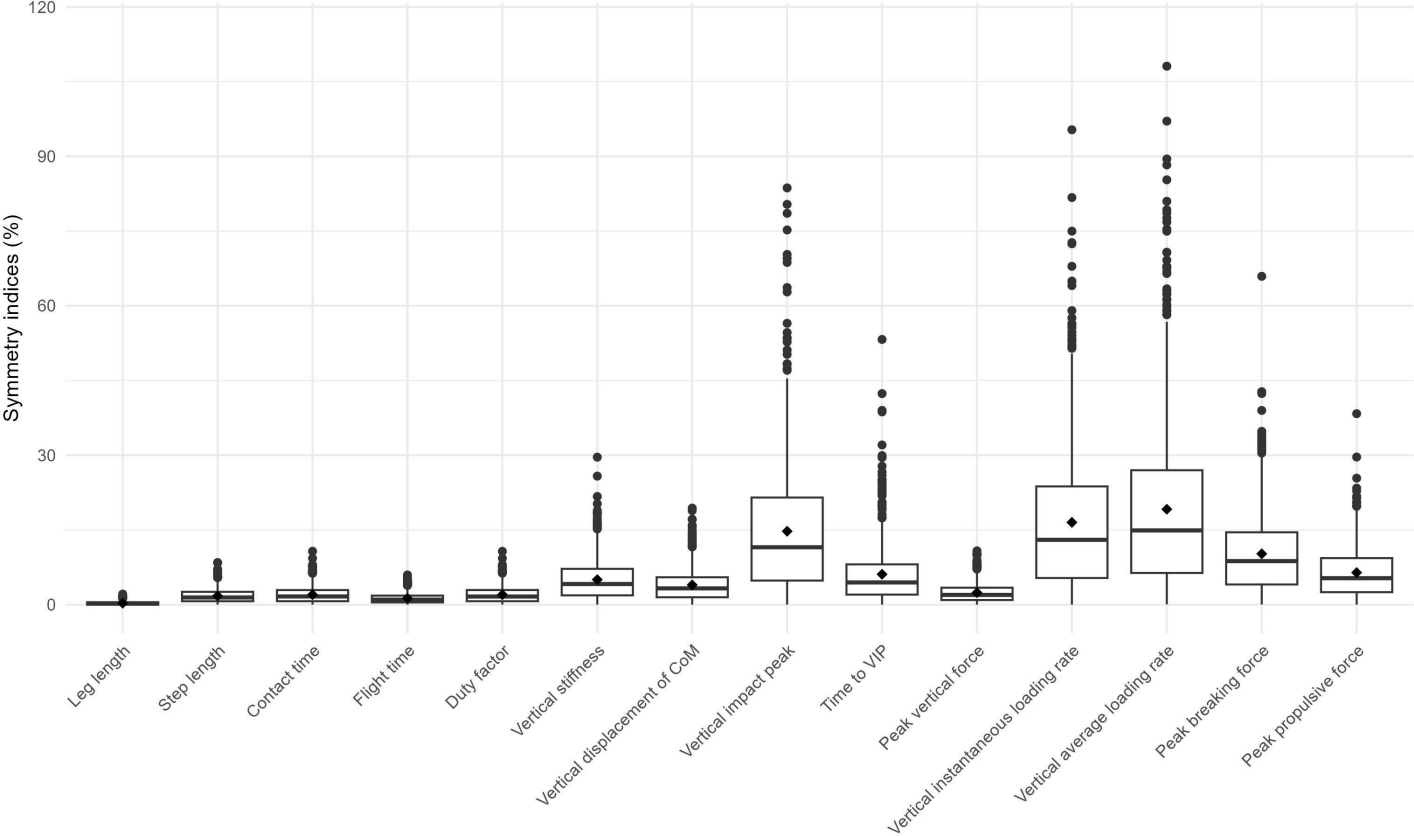
Box plots of the symmetry indices for each spatiotemporal and ground reaction force characteristic investigated. The lower and upper box boundaries indicate the 25th and 75th percentiles, respectively; the middle line inside the box represents the median, and the filled diamond is the mean. Whiskers below and above the boxes represent the 25th percentile−1.5×IQR and 75th percentile+1.5×IQR, respectively. CoM, centre of mass; VIP, vertical impact peak.

Table 1 presents the descriptive statistics of the 836 participants analysed. Age was 40.3 years (SD 10.0), 38.6% were women and body mass index was 24.0 (SD 2.9). Asymmetry was 0.2% (IQR: 0%–0.5%) for leg length and ranged from 1.0% (flight time) to 15.0% (vertical average loading rate) for the biomechanical variables, with overall greater values among the kinetic variables (figure 1). Online supplemental material S2 presents the correlation matrix among asymmetries in the spatiotemporal and kinetic variables.

**Table 1:** Descriptive statistics of the participants (n=836)

Characteristics	Unit/qualifier	All participants
Participants’ characteristics		
Age	Years	40.3±9.9
Sex	Male	513 (61.4%)
	Female	323 (38.6%)
Height	cm	174±9
Body mass	kg	73.0±12.3
Body mass index	kg/m^2^	24.0±2.9
Proportion of fat mass	%	22.4±7.2
Previous Injury	No	700 (83.7%)
	Yes	136 (16.3%)
Running experience	Years	6 (3–13)
Regularity (last 12 months)	Months	12 (6–12)
Running participation (follow-up)		
Running frequency	Sessions per week	1.4 (1.0–2.0)
Mean session duration	min	50 (40–61)
Mean session distance	km	8.0 (6.0–10.0)
Mean session intensity	a.u.	3.8 (3.1–4.4)
Mean speed	km/hour	9.7 (8.8–10.6)
Symmetry Index		
Steps analysed	n	316±49
Leg length	%	0.2 (0–0.5)
Step length	%	1.4 (0.7–2.6)
Contact time	%	1.7 (0.7–2.9)
Flight time	%	1.0 (0.5–1.8)
Duty factor	%	1.6 (0.7–2.9)
Vertical displacement of CoM	%	3.3 (1.5–5.5)
Vertical stiffness	%	4.1 (1.9–7.2)
Peak vertical force	%	2.0 (0.9–3.4)
Peak breaking force	%	8.7 (4.1–14.6)
Peak propulsive force	%	5.3 (2.5–9.4)
VIP	%	11.5 (4.8–21.5)
Time to VIP	%	4.5 (2.0–8.1)
Vertical instantaneous loading rate	%	13.0 (5.3–23.8)
Vertical average loading rate	%	15.0 (6.4–27.1)

A non-normally distributed variable is presented as median (IQR).

a.u., arbitrary unit; CoM, the centre of mass; VIP, vertical impact peak.

### Injuries

During follow-up, 218 participants reported at least one injury. For 107 participants, the first injury met the running-related injury definition. For 111, the injury was defined as a competing injury (including running-related injuries not associated with one of the limbs, eg, lower back). Running-related injury incidence was 4.8 per 1000 hour of running (95% CI: 4.0 to 5.8). Additional information on running-related injuries is presented in online supplemental material S3.

### Determinants of asymmetry

The correlations between the potential determinants and asymmetry in biomechanical variables were negligible, as the coefficients ranged from 0.01 to 0.13 (online supplemental material S4). Age was not significantly correlated with any Symmetry Index. Body mass was highly correlated with height (r>70) and only marginally correlated with two symmetry indices, so body mass was not included in the linear regressions. Table 2 presents the multiple linear regression models for each biomechanical variable. The adjusted R^2^ of the regression models were very low (<0.01 to 0.03), indicating that the variance of asymmetry is only marginally explained by the determinants investigated here.

**Table 2:** Determinants of asymmetry (n=836)

Symmetry Index	Leg length (SI)	Sex	Height	Fat mass	Previous injury	Running experience	Running regularity (last 12 months)	Shoe version	Running speed	Adjusted R^2^
Step length	−0.22 (−0.47 to 0.04)		−1.06 (−2.23 to 0.11)	−0.02 (−0.03 to −0.01)				−0.24 (−0.42 to −0.06)		*0.02*
	*0.095*		*0.077*	***0.005***				***0.011***		
Contact time		0.27 (−0.04 to 0.57)		−0.04 (−0.06 to −0.02)						*0.02*
		*0.085*		***<0.001***						
Flight time	0.21 (0.01 to 0.40)			−0.02 (−0.03 to −0.01)					−0.11 (−0.18 to −0.05)	*0.02*
	***0.043***			***0.003***					***<0.001***	
Duty factor	0.26 (−0.04 to 0.57)	0.27 (−0.04 to 0.57)		−0.04 (−0.06 to −0.02)						*0.02*
	*0.093*	*0.088*		***<0.001***						
Vertical displacement of CoM	0.82 (0.23 to 1.41)	−0.94 (−1.53 to −0.34)	−3.53 (−6.86 to −0.19)							*0.02*
	***0.006***	***0.002***	***0.038***							
Vertical stiffness				−0.04 (−0.07 to 0.00)			0.07 (0.01 to 0.13)			*0.01*
				***0.064***			***0.032***			
Peak vertical force	0.64 (0.28 to 1.01)	−0.66 (−1.02 to −0.28)	−2.60 (−4.67 to −0.52)			0.02 (0.00 to 0.03)		−0.22 (−0.49 to 0.004)		*0.03*
	***0.001***	***0.001***	***0.014***			***0.045***		*0.090*		
Peak breaking force	2.77 (1.28 to 4.26)			−0.09 (−0.16 to −0.01)						*0.02*
	***<0.001***			***0.022***						
Peak propulsive force						0.04 (−0.01 to 0.08)				*<0.01*
						*0.086*				
VIP			−13.7 (−23.8 to −3.7)							*0.01*
			***0.007***							
Time to VIP										*<0.01*
Vertical instantaneous loading rate	*2.19 (−0.41 to 4.78*)		*−12.1 (−22.9 to −1.3*)					*3.30 (1.43 to 5.17*)		*0.02*
	*0.098*		***0.028***					***0.001***		
Vertical average loading rate									*−1.00 (−1.83 to −0.16*)	*0.01*
									***0.020***	

Values are coefficients (95% CI). P values are presented in italics. P values<0.05 are presented in bold.

CoM, centre of mass; SI, Symmetry Index; VIP, vertical impact peak.

### Injury risk

Table 3 presents the estimates for the subhazard rate ratios for asymmetry in each biomechanical variable. In the crude analysis (Model 1), greater asymmetry for flight time and peak braking force was associated with lower injury risk. Adjustment for potential confounders (Model 2, online supplemental material S5) and mutual adjustment for all biomechanical variables (Model 3) marginally influenced the estimates, thus confirming the main findings from Model 1. After stratification by shoe version, greater asymmetry for flight time and peak breaking force remained associated with lower injury risk in runners who received the soft shoe version.

**Table 3:** Association between asymmetry and injury risk (n=848)

Symmetry Index	Model 1	Model 2	Model 3	Model 4	
				Soft	Hard
Leg length	1.14 (0.73 to 1.80)	1.09 (0.69 to 1.72)	–	–	–
	*0.566*	*0.703*			
Step length	0.90 (0.77 to 1.05)	0.90 (0.77 to 1.05)	–	–	–
	*0.177*	*0.167*			
Contact time	0.88 (0.76 to 1.02)	0.87 (0.75 to 1.01)	–	–	–
	*0.087*	*0.069*			
Flight time	0.80 (0.64 to 1.00)	0.79 (0.63 to 1.00)	0.80 (0.64 to 0.99)	0.67 (0.50 to 0.89)	–
	***0.048***	***0.048***	***0.045***	***0.006***	
Duty factor	0.88 (0.76 to 1.02)	0.87 (0.75 to 1.01)	–	–	–
	*0.089*	*0.071*			
Vertical displacement of CoM	0.95 (0.89 to 1.02)	0.95 (0.88 to 1.02)	–	–	–
	*0.147*	*0.127*			
Vertical stiffness	0.95 (0.91 to 1.01)	0.95 (0.90 to 1.00)	–	–	–
	*0.082*	*0.053*			
Peak vertical force	0.96 (0.88 to 1.06)	0.96 (0.88 to 1.06)	–	–	–
	*0.463*	*0.419*			
Peak breaking force	0.96 (0.93 to 0.99)	0.96 (0.93 to 0.98)	0.96 (0.93 to 0.98)	0.95 (0.91 to 0.99)	0.97 (0.93 to 1.00)
	***0.002***	***0.002***	***0.002***	***0.009***	*0.083*
Peak propulsive force	0.99 (0.95 to 1.03)	0.99 (0.95 to 1.03)	–	–	–
	*0.567*	*0.570*			
VIP	1.00 (0.98 to 1.01)	1.00 (0.98 to 1.01)	–	–	–
	*0.467*	*0.635*			
Time to VIP	0.97 (0.93 to 1.01)	0.97 (0.94 to 1.01)	–	–	–
	*0.198*	*0.212*			
Vertical instantaneous loading rate	*1.00 (0.99 to 1.01*)	*1.00 (0.99 to 1.01*)	–	–	–
	*0.974*	*0.905*			
Vertical average loading rate	*0.99 (0.98 to 1.00*)	*0.99 (0.98 to 1.00*)	–	–	–
	*0.111*	*0.137*			

Running exposure: 22 178 hours. A total of 107 running-related overuse injuries were related to the lower limb. Values are subhazard rate ratio (95% CI). P values are presented in italics. P values<0.05 are presented in bold. Model 1 is the unadjusted model. Model 2=Model 1 adjusted for previous injury and running speed. Model 3=Model 2 mutually adjusted for all symmetry indices. Model 4=Model 3 stratified by shoe version (ie, soft and hard).

CoM, centre of mass; VIP, vertical impact peak.

### Limb comparisons in injured runners

No significant difference was observed at baseline in spatiotemporal and kinetic variables between the involved and uninvolved limbs in runners who had sustained an injury (table 4).

**Table 4:** Baseline comparison in spatiotemporal and kinetic variables between the involved and uninvolved limbs in runners who have sustained an injury (n=107)

Variables	Unit	Injured limb	Non-injured limb	Mean difference	P value	Cohen’s d
Leg length	m	0.91±0.06	0.91±0.06	9×10^−6^	0.914	<0.01
Step length	m	1.03±0.13	1.03±0.13	3×10^−4^	0.914	<0.01
Contact time	ms	280±30	279±31	−0.2	0.914	0.01
Flight time	ms	450±35	450±35	0.2	0.914	0.01
Duty factor	%	38.3±3.3	38.3±3.4	−0.03	0.914	0.01
Vertical displacement of CoM	mm	78.2±11.8	78.6±11.5	0.37	0.756	0.03
Vertical stiffness	kN/m	38.1±5.3	37.9±5.3	−0.22	0.756	0.04
Peak vertical force	BW	2.26±0.21	2.27±0.20	0.01	0.621	0.05
Peak breaking force	BW	0.33±0.06	0.33±0.06	0.001	0.914	0.02
Peak propulsive force	BW	−0.26±0.06	−0.26±0.05	−4×10^−4^	0.914	0.01
VIP	BW	0.43±0.13	0.44±0.14	0.008	0.756	0.06
Time to VIP	ms	36.4±5.7	35.9±5.4	−0.5	0.518	0.10
VILR	BW/s	32.6±10.6	33.1±10.4	0.50	0.880	0.05
VALR	BW/s	17.3±7.2	17.9±7.3	0.63	0.621	0.09

P values are adjusted according to Benjamini-Hochberg.^25^ Values are mean±SD.

BW, body weight; CoM, centre of mass VALR, vertical average loading rate; VILR, vertical instantaneous loading rate; VIP, vertical impact peak.

## Discussion

This study aimed to describe the magnitude of asymmetry of spatiotemporal and kinetic variables in 800+ recreational runners, identify the determinants of asymmetry, investigate the association with injury risk and compare the involved and uninvolved limbs in runners who have sustained an injury during follow-up. The main finding is that gait asymmetry was not associated with higher injury risk for investigated spatiotemporal and kinetic variables. Conversely, a lower risk was observed in runners having greater asymmetry in flight time and peak breaking force (table 3).

### Variability in asymmetry

While craniocaudal and dorsoventral asymmetries are commonly accepted as unproblematic, bilateral asymmetry (a broad term referring to bilateral trunk asymmetries and interlimb asymmetries) triggers more debates and comes with deeply held beliefs in sports medicine.^27^ Yet, human anatomy is abundant with bilateral asymmetries, especially in thoracic and abdominal organs.^28^ Anatomical bilateral asymmetries extend to the lower limb as well, for instance, in the hamstrings’ structure and functionality,^29^ which suggests that bilaterally cyclic activities such as running should result in interlimb asymmetries in biomechanics in healthy populations.^7^ Indeed, our findings in recreational runners align with previous studies reporting high levels of asymmetry in different populations of healthy runners, with large interindividual variability.^9 11^

The magnitude of asymmetry varied across the variables investigated. Globally, kinetic variables showed more asymmetry than spatiotemporal variables (figure 1), which is consistent with previous work.^5–7^ In this study, only vertical impact peak and vertical loading rate exceeded 10%, while spatiotemporal variables were below 2%. Based on 800+ recreational runners, this observation provides additional substantial evidence that asymmetry is variable dependent and calls for caution when applying arbitrary thresholds to determine ‘acceptable’ between-limb differences.

### Determinants of asymmetry

To the best of our knowledge, this is the first study investigating such a large set of potential determinants of asymmetry. Only negligible correlations were found (online supplemental material S4), explaining only marginal between-subject variability in asymmetry. Fat mass proportion and leg length asymmetry are the variables most frequently associated with asymmetry. As expected,^4^ leg length asymmetry was associated with greater asymmetry in several variables (table 2). There is some evidence that sex has no or very limited effect on asymmetry in runners.^7 11^ Consistently, sex was only identified as a determinant for two variables in this study. Running speed was only associated with lower asymmetry for flight time, which is consistent with previous work on within-subject effects, suggesting that asymmetry does not vary with speed.^5 7 11^ Thus, it seems that between-subject and within-subject variations in running speed have a limited impact on asymmetry. Surprisingly, previous injury was not identified as a determinant of asymmetry, while a retrospective study showed greater asymmetry in runners with previous injury.^3^ Also, peak vertical shock (tibial acceleration) was 15% higher in the involved limb as compared with the uninvolved limb in a group of runners with a history of stress fractures.^9^ The time elapsed since the previous injury may explain the research discrepancies and deserve further analysis.

### Injury risk

Previous studies reported no difference in asymmetry between never-injured and previously injured runners, but they found higher loading on the injured limb (eg, peak tibial shock).^9 10^ However, these studies were retrospective, and limb loading differences may be the result instead of the cause of injury. Only two prospective studies have investigated interlimb asymmetry related to injury risk. In cross-country runners, there were no differences in asymmetry of kinematic and kinetic variables between runners who sustained an injury and those who remained healthy during follow-up.^5^ In novice runners, asymmetry in impact peak at baseline was lower in runners who sustained an injury during follow-up.^11^ Furthermore, no association with injury was found when using time-to-event analyses, the recommended approach to investigate injury aetiology.^30^ Consistently, the present study did not reveal any association between asymmetry in spatiotemporal and kinetic variables and greater injury risk. Conversely, lower injury risk was found in runners with greater asymmetry for flight time and peak breaking force. Notably, the findings were rather consistent through the four regression models. One may speculate that the protective effect of greater asymmetry in flight time and peak braking force represents a necessary adaptation of the runner’s technique to the individual morphology to limit injury risk.

### Clinical implications

The search for interlimb asymmetry as a risk factor for injury in running seems to originate more from popular beliefs than scientific evidence. None of the three prospective studies on asymmetry in running biomechanics and injury risk provided evidence that asymmetry predisposes runners to higher injury risk, as hypothesised in the literature.^3^ Furthermore, following previous work, no differences in loading between the injured and non-injured leg were found in this study.^11^ Therefore, the search for gait asymmetry in the lower limbs in healthy recreational runners as an approach for primary prevention is not supported by scientific evidence. Given the large variation in asymmetry levels across the variables investigated, applying a threshold to determine ‘acceptable’ or ‘normal’ asymmetry levels appears challenging.^6 11^

### Strengths and limitations

This is the largest (and one of the few) prospective studies on the association between asymmetry in spatiotemporal and kinetic variables and injury risk in recreational runners. Furthermore, the reliability of the measurements is strengthened by the large number of steps analysed per participant’s leg (158±25 steps) and the use of frequency-domain analyses to assess impact loading.^20 21^ However, this study also has some limitations. First, the test was performed on an instrumented treadmill. Although there is some evidence that there are no clinically relevant differences in spatiotemporal and kinetic variables on treadmill compared with overground running,^31^ future research should investigate if levels of asymmetry are comparable, as speed fluctuations are very limited on a treadmill. Second, asymmetry in kinematics was not investigated. Previous work reported that asymmetries in joint moments and work tended to be the largest,^7^ but their association with injury risk is also worth examining. Further prospective studies investigating running kinematics are needed to confirm that asymmetry in running biomechanics does not lead to injury.

## Conclusion

This study demonstrated that asymmetry in spatiotemporal and kinetic variables was not associated with higher injury risk in recreational runners. The study also illustrated the high intraindividual (across variables) and interindividual variability of asymmetry in biomechanics. The determinants identified in this study only marginally explained the variability in asymmetry.

## Data Availability

Data are available upon reasonable request. Individual mean values for each spatiotemporal and kinetic variable can be requested for research purposes only via email to the corresponding author.
