# Supplementary material for: Gait asymmetry in spatiotemporal and kinetic variables does not increase running-related injury risk in lower limbs: a secondary analysis of a randomised trial including 800+ recreational runners
Source: BMJ Open Sport Exerc Med. 2024 Jan 5;10(1):e001787. doi: 10.1136/bmjsem-2023-001787 (PMC10773390; doi:10.1136/bmjsem-2023-001787)

SUPPLEMENTAL MATERIAL

Supplemental Material S1: Flow chart

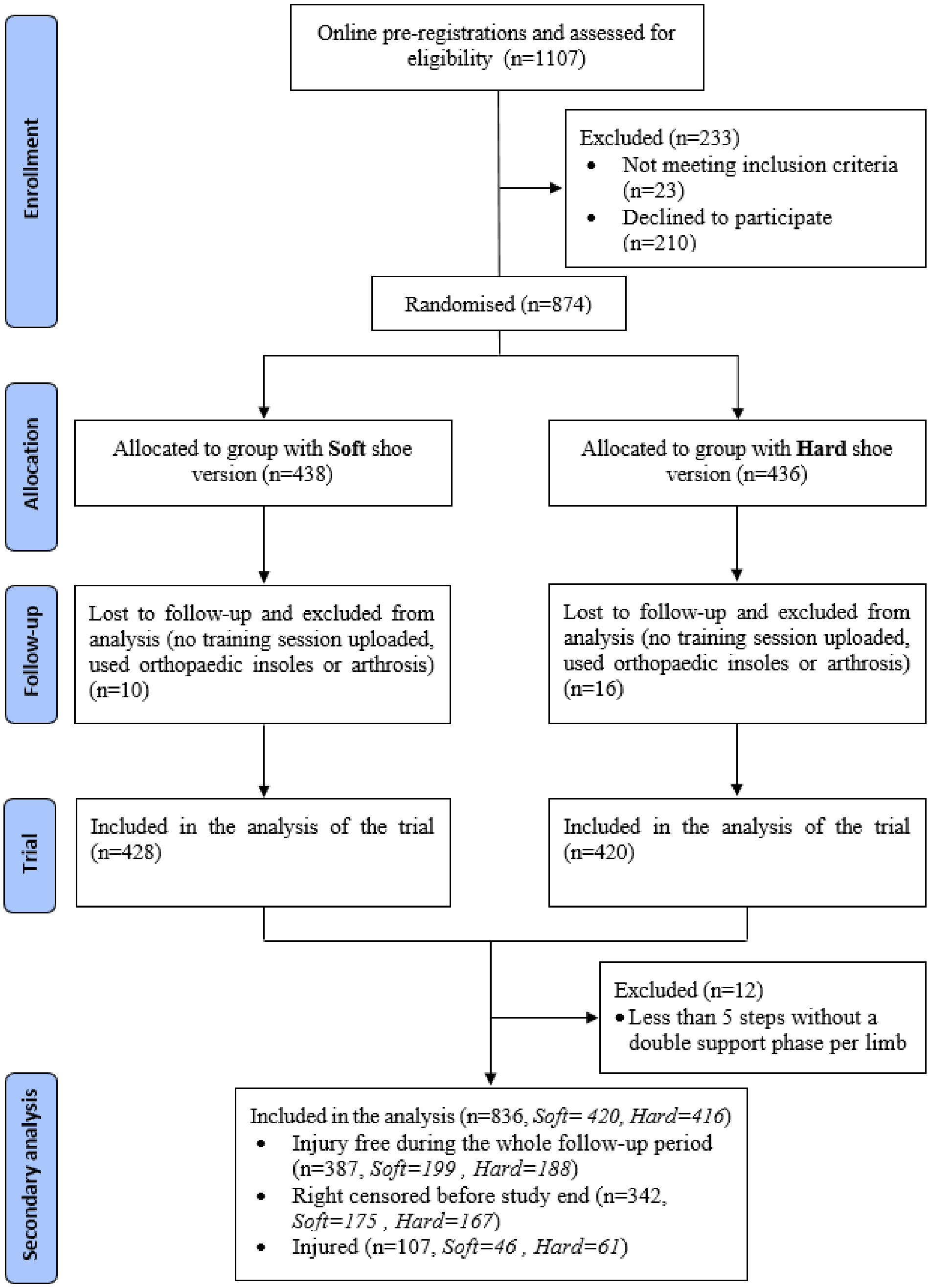

Supplemental Material S2: Correlation matrix between asymmetries in the spatiotemporal and kinetic variables (n=836).

	Step length	Contact time	Flight time	Duty factor	Vertical displ. of CoM	Vertical Stiffness	Peak vertical force	Peak breaking force	Peak propulsive force	VIP	Time to VIP	Vertical instant. loading rate	Vertical average loading rate
Step length	1.00												
Contact time	0.32<br>***<0.001***	1.00											
Flight time	0.31<br>***<0.001***	0.97<br>***<0.001***	1.00										
Duty factor	0.31<br>***<0.001***	0.99<br>***<0.001***	0.97<br>***<0.001***	1.00									
Vertical displ. of CoM	0.27<br>***<0.001***	0.02<br>*0.645*	0.03<br>*0.373*	0.2<br>*0.665*	1.00								
Vertical Stiffness	0.11<br>***0.001***	0.13<br>***<0.001***	0.12<br>***<0.001***	0.13<br>***<0.001***	0.52<br>***<0.001***	1.00							
Peak vertical force	0.04<br>*0.288*	0.32<br>***<0.001***	0.30<br>***<0.001***	0.32<br>***<0.001***	0.35<br>***<0.001***	0.14<br>***<0.001***	1.00						
Peak breaking force	0.02<br>*0.479*	0.01<br>*0.748*	-0.01<br>*0.872*	0.01<br>*0.729*	0.11<br>***<0.001***	0.13<br>***<0.001***	0.03<br>*0.467*	1.00					
Peak propulsive force	0.05<br>*0.167*	0.08<br>***0.024***	0.09<br>***0.014***	0.08<br>***0.024***	0.13<br>***<0.001***	0.15<br>***<0.001***	0.10<br>***0.004***	0.10<br>***0.006***	1.00				
VIP	0.10<br>***0.005***	0.06<br>*0.068*	0.06<br>*0.073*	0.06<br>*0.074*	0.01<br>*0.803*	0.06<br>*0.107*	-0.01<br>*0.979*	0.15<br>***<0.001***	0.06<br>*0.080*	1.00			
Time to VIP	0.17<br>***<0.001***	0.17<br>***<0.001***	0.16<br>***<0.001***	0.17<br>***<0.001***	0.01<br>*0.777*	0.03<br>*0.410*	0.09<br>***0.009***	0.11<br>***0.001***	0.11<br>***0.002***	0.23<br>***<0.001***	1.00		
Vertical instant. loading rate	0.06<br>*0.069*	0.06<br>*0.090*	0.07<br>***0.034***	0.06<br>*0.092*	0.08<br>***0.016***	0.09<br>***0.010***	-0.01<br>*0.686*	0.14<br>***<0.001***	0.10<br>***0.003***	0.69<br>***<0.001***	0.22<br>***<0.001***	1.00	
Vertical average loading rate	0.14<br>***<0.001***	0.02<br>*0.548*	0.02<br>*0.513*	0.02<br>*0.566*	0.01<br>*0.815*	0.03<br>*0.341*	-0.02<br>*0.488*	0.10<br>***0.005***	0.04<br>***0.267***	0.63<br>***<0.001***	0.57<br>***<0.001***	0.49<br>***<0.001***	1.00

P-values are presented in *Italic*; P-values <0.05 are presented in Bold; CoM: Center of Mass; VIP: Vertical Impact Peak

BMJ Publishing Group Limited (BMJ) disclaims all liability and responsibility arising from any reliance placed on this supplemental material which has been supplied by the author(s)

Supplemental Material S3: Characteristics of self-reported running-related injuries (n=107).

	All participants	
	N	%
Injury location		
Buttock / Pelvis	6	5.6
Hip/groin	3	2.8
Thigh	8	7.5
Knee	26	24.3
Lower leg	23	21.5
Ankle	22	20.6
Foot	19	17.7
Injury type		
Tendon	63	58.9
Muscle	21	19.6
Capsules and ligaments	5	4.7
Bone structures	4	3.7
Other overuse injuries	14	13.1
Injury severity		
Moderate (8-28 days)	68	63.6
Major (>28 days)	39	36.4
Context		
Training	102	95.3
Competition	5	4.7

Only running-related overuse injuries to the lower limb are included (running-related injuries not associated to one of the limbs, such as lower back injuries, are considered as competing injuries).

Supplemental Material S4: Correlation between personal characteristics and asymmetry in spatiotemporal and kinetic variables (n=836).

	Leg length SI	Sex	Age	Height	Body mass	Fat mass	Prev. inj	Run. exp.	Run. reg.	Shoe version	Run. speed
Step length	-0.06	-0.05	-0.04	-0.02	-0.02	-0.08	0.02	-0.01	0.01	-0.09	0.01
	0.090	*0.168*	*0.292*	*0.585*	*0.540*	***0.027***	*0.514*	*0.852*	*0.827*	***0.010***	*0.930*
Contact time	0.06	-0.04	-0.05	0.02	-0.07	-0.13	0.03	-0.01	0.048	-0.01	0.04
	0.106	*0.211*	*0.141*	*0.570*	***0.044***	***<0.001***	*0.415*	*0.843*	*0.170*	*0.845*	*0.204*
Flight time	0.07	0.01	-0.01	0.01	-0.04	-0.03	0.02	-0.01	0.01	-0.01	-0.08
	0.056	*0.803*	*0.804*	*0.912*	*0.216*	*0.333*	*0.592*	*0.859*	*0.729*	*0.750*	***0.029***
Duty factor	0.06	-0.04	-0.05	0.02	-0.07	-0.13	0.03	-0.01	0.05	-0.01	0.04
	0.098	*0.205*	*0.146*	*0.557*	***0.044***	***<0.001***	*0.401*	*0.851*	*0.164*	*0.858*	*0.202*
Vertical displ. of CoM	0.10	-0.08	0.03	-0.01	0.06	-0.01	0.07	0.05	-0.01	-0.03	-0.01
	0.005	***0.026***	*0.329*	*0.922*	*0.076*	*0.689*	*0.059*	*0.139*	*0.703*	*0.457*	*0.991*
Vertical Stiffness	0.03	-0.06	0.02	-0.01	-0.01	-0.08	0.06	0.06	0.09	0.01	0.07
	0.386	*0.073*	*0.565*	*0.940*	*0.818*	***0.024***	*0.109*	*0.061*	***0.012***	*0.803*	***0.041***
Peak vertical force	0.12	-0.09	0.02	-0.01	0.01	-0.09	0.02	0.08	0.01	-0.05	0.09
	0.001	***0.008***	*0.506*	*0.888*	*0.979*	***0.008***	*0.621*	***0.002***	*0.944*	*0.139*	***0.012***
Peak breaking force	0.12	-0.05	0.03	0.03	-0.01	-0.08	-0.01	0.02	0.02	-0.05	0.06
	<0.001	*0.147*	*0.386*	*0.435*	*0.728*	***0.025***	*0.667*	*0.500*	*0.657*	*0.193*	*0.080*
Peak propulsive force	0.05	-0.01	0.05	-0.04	-0.05	-0.03	0.04	0.06	-0.03	0.05	-0.02
	0.167	*0.925*	*0.149*	*0.212*	*0.193*	*0.449*	*0.258*	*0.086*	*0.332*	*0.173*	*0.553*
VIP	0.2	0.04	-0.01	-0.09	-0.03	0.02	-0.06	-0.03	-0.04	0.05	-0.08
	0.477	*0.213*	*0.944*	***0.007***	*0.346*	*0.549*	*0.084*	*0.382*	*0.284*	*0.154*	***0.016***
Time to VIP	0.2	0.04	-0.02	-0.04	-0.04	0.01	0.01	0.03	-0.02	-0.01	0.01
	0.598	*0.306*	*0.593*	*0.253*	*0.198*	*0.970*	*0.718*	*0.432*	*0.532*	*0.722*	*0.978*
Vertical instant. loading rate	0.7	0.05	-0.03	-0.08	-0.04	0.02	0.01	-0.01	-0.04	0.12	-0.07
	0.059	*0.122*	*0.319*	***0.023***	*0.253*	*0.582*	*0.908*	*0.722*	*0.202*	***<0.001***	***0.034***
Vertical average loading rate	-0.01	0.02	0.01	-0.06	0.01	0.04	0.01	0.01	-0.07	0.03	-0.08
	0.702	*0.635*	*0.808*	*0.067*	*0.718*	*0.290*	*0.857*	*0.817*	**0.036**	*0.445*	***0.020***

P-values are presented in *Italic*; P-values <0.05 are presented in Bold; CoM: Center of Mass; VIP: Vertical Impact Peak; Prev. Inj.: Previous injury; Run. exp.: Running experience; Run. reg.: Running regularity (last 12 months); SI: Symmetry Index.

Supplemental Material S5: Directed Acyclic Graph (DAG) representing the causal pathways between symmetry indices and running-related injury (RRI). Created on http://dagitty.net. The outcome variable is identified as a blue oval with black outline and the main exposure is identified as green ovals with black outline. Variables on the causal pathways are identified as blue ovals, determinants of the exposure as green ovals and potential confounders as red ovals.

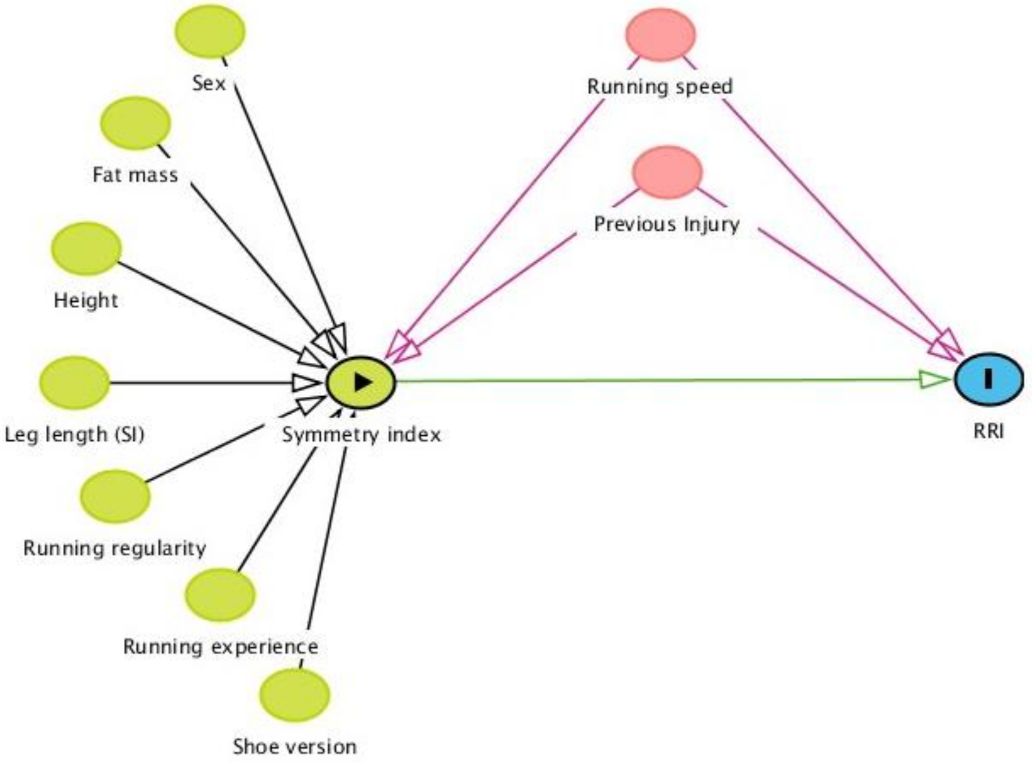

Supplement: Supplementary data [file bmjsem-2023-001787supp001.pdf]
